# Endoscopic Removal of Gastrointestinal Stromal Tumors in the Stomach: A Single-Center Experience

**DOI:** 10.1155/2019/3087298

**Published:** 2019-10-22

**Authors:** Yingjie Guo, Xue Jing, Jian Zhang, Xueli Ding, Xiaoyu Li, Tao Mao, Zibin Tian

**Affiliations:** ^1^Department of Gastroenterology, The Affiliated Hospital of QingDao University, Qingdao, 266003 Shandong Province, China; ^2^Department of General Surgery, The Affiliated Hospital of QingDao University, Qingdao, 266003 Shandong Province, China

## Abstract

**Background and Aims:**

Endoscopic removal of GISTs (gastrointestinal stromal tumors) is recently recognized, but less is known about its efficacy and safety. This study is aimed at assessing the feasibility, clinical efficacy, and safety of the endoscopic removal of gastric GISTs.

**Patients and Methods:**

Endoscopic removal (ER) of GISTs was performed in 134 patients at our hospital between January 2015 and January 2019. The clinical features, surgical outcomes, complications, pathological diagnosis, and risk classification were evaluated retrospectively.

**Results:**

ER was successful in 131 cases (98%), including 58 by ESD (endoscopic submucosal dissection), 43 by ESE (endoscopic submucosal excavation), 25 by EFTR (endoscopic full-thickness resection), and 5 by STER (submucosal tunneling endoscopic resection). In addition, GISTs of two cases were resected using LECS (laparoscopic and luminal endoscopic cooperative surgery) for the extraluminal and intraluminal growth pattern. The average tumor size was 1.89 ± 1.25 cm (range: 0.5-6.0 cm). Of these patients, 26 cases had a large tumor size (range: 2.0-6.0 cm), and endoscopic removal was successful in all of them. During the procedure, endoclips were used to close the perforation in all cases, without conversion to open surgery. The average length of hospital stay was 5.50 ± 2.15 days (range: 3-10 days). In the risk classification, 106 (79.7%) were of a very low risk, 25 (18.8%) of a low risk, and 2 (1.5%) of a moderate risk. The moderate-risk cases were treated with imatinib mesylate after ER. No recurrence or metastasis was observed during the follow-up period of 23 ± 8 months (range: 3-48 months).

**Conclusions:**

The endoscopic treatment is feasible, effective, and safe for gastric GISTs, and individualized choice of approaches is recommended for GISTs.

## 1. Introduction

GISTs (gastrointestinal stromal tumors) are the most common mesenchymal neoplasms of the gastrointestinal tract [1, 2]. GISTs are common in the stomach (60-70%), and most primary GISTs potentially become malignancies [3, 4]. According to the National Comprehensive Cancer Network, surgical removal is recommended for GISTs larger than 2 cm in size, and either surgical removal or surveillance is advised for those smaller than 2 cm [5]. However, the poor compliance of patients may contribute to delayed diagnosis of malignancies and treatment. It has been indicated that even small GISTs (<2.0 cm) which have a high mitotic index are potentially malignant [6, 7]. Additionally, long-term survival with tumors may bring a great psychological burden to most patients. Thus, it is believed that it is crucial to diagnose and treat GISTs at an early stage.

EUS (endoscopic ultrasonography) and endoscopy have a great advantage for early diagnosis and treatment of GISTs [8, 9]. ER (endoscopic removal) is applied to not only mucous layer tumors but also submucosal tumors, e.g., GISTs in the muscularis propria (MP) layer. Endoscopic methods include ESD (endoscopic submucosal dissection), EFTR (endoscopic full-thickness resection), ESE (endoscopic submucosal excavation), STER (submucosal tunneling endoscopic resection), and LECS (laparoscopic and luminal endoscopic cooperative surgery) [10]. Recently, some studies with a small sample size reported that ER was successful in gastric GISTs. However, there are limited data on the feasibility and safety of ER in gastric GISTs, especially those with a large tumor size. In recent years, this technique has been applied to gastric GISTs in our center, and good therapeutic outcomes have been achieved. This study is aimed at assessing the feasibility, efficacy, and safety of ER in GISTs at the muscularis propria layer, so as to guide clinical treatment of GISTs.

## 2. Patients and Methods

### 2.1. Subjects

**Figure 1:**
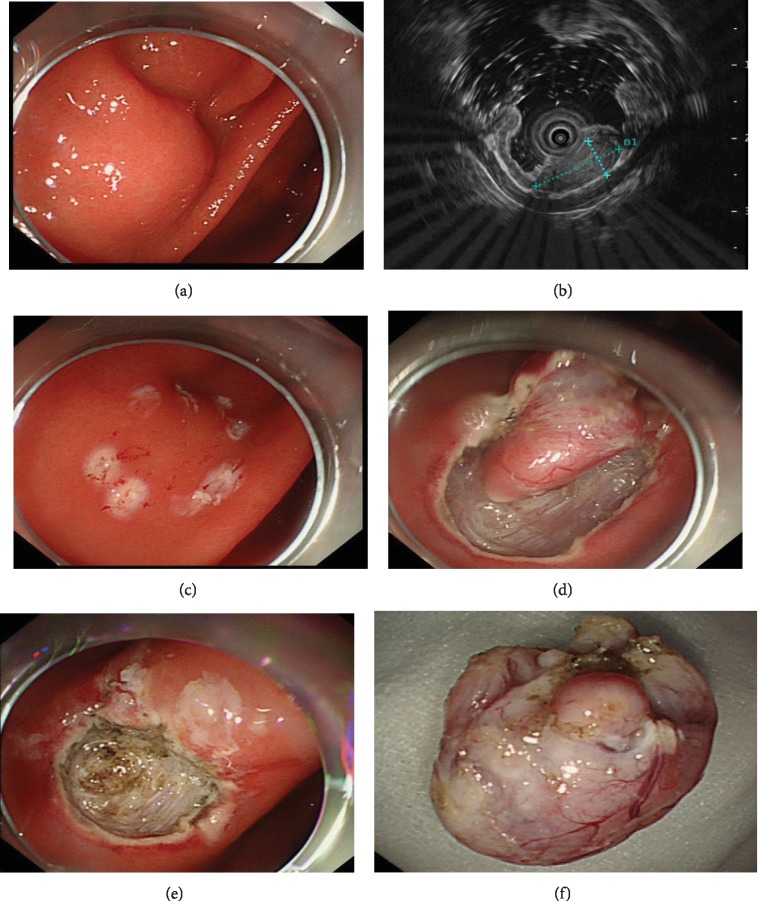
Endoscopic submucosal dissection of a gastric GIST. (a) A gastric GIST is observed. (b) The tumor originates from the muscularis propria layer on EUS. (c, d) After making dots, submucosal dissection of the tumor is performed using an IT knife. (e) The lesion is removed completely. (f) View of the tumor after resection.

The clinical data of patients who accepted ER at the Affiliated Hospital of Qingdao University (Qingdao, China) between January 2015 and January 2019 were retrospectively analyzed. Of them, 134 patients who underwent ER of gastric GISTs were identified (Figure 1). Metastasis was excluded by ultrasonography and/or CT scanning of the abdomen before surgery in all patients. EUS was carried out to determine the growth pattern, the layer of origin, and the exact tumor size before ER. All patients were informed of the procedure and received detailed explanations about the risks and benefits of ER, and the informed consent was signed before ER. The study protocol was reviewed and approved by the Institutional Review Board of the Affiliated Hospital of Qingdao University.

### 2.2. Endoscopic Procedures

**Figure 2:**
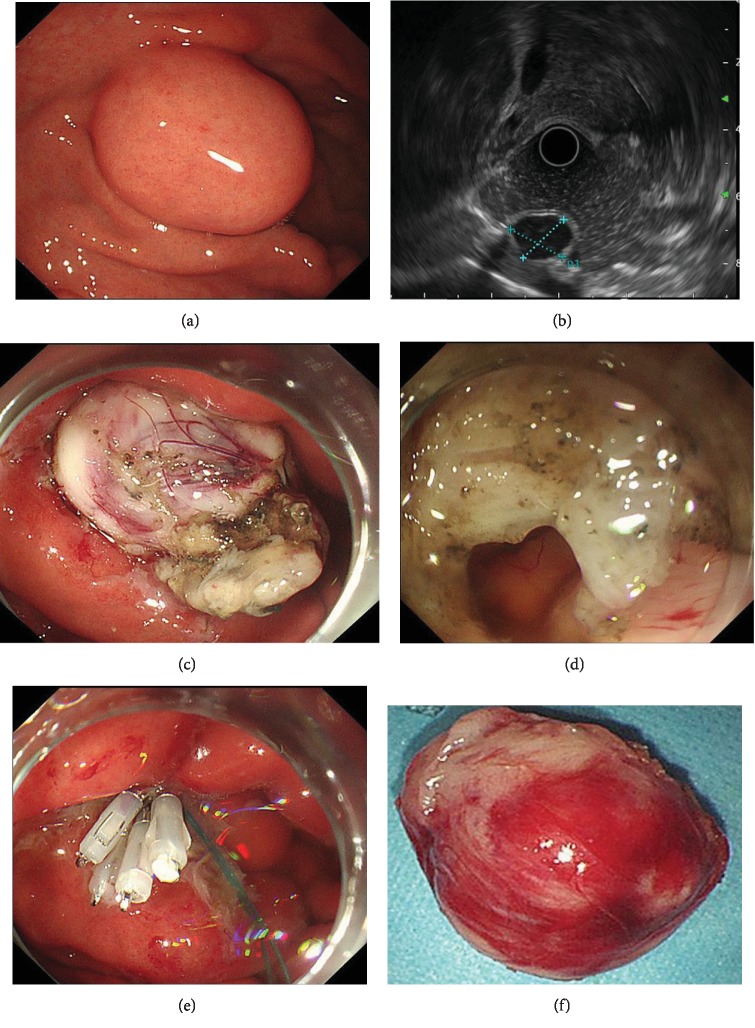
Endoscopic full-thickness resection of a gastric GIST. (a) A gastric GIST is observed. (b) The tumor originates from the muscularis propria layer on EUS. (c, d) Submucosal dissection of the tumor is performed using an IT knife. (e) The wound was closed with a nylon band and several clips. (f) View of the tumor after resection.

**Figure 3:**
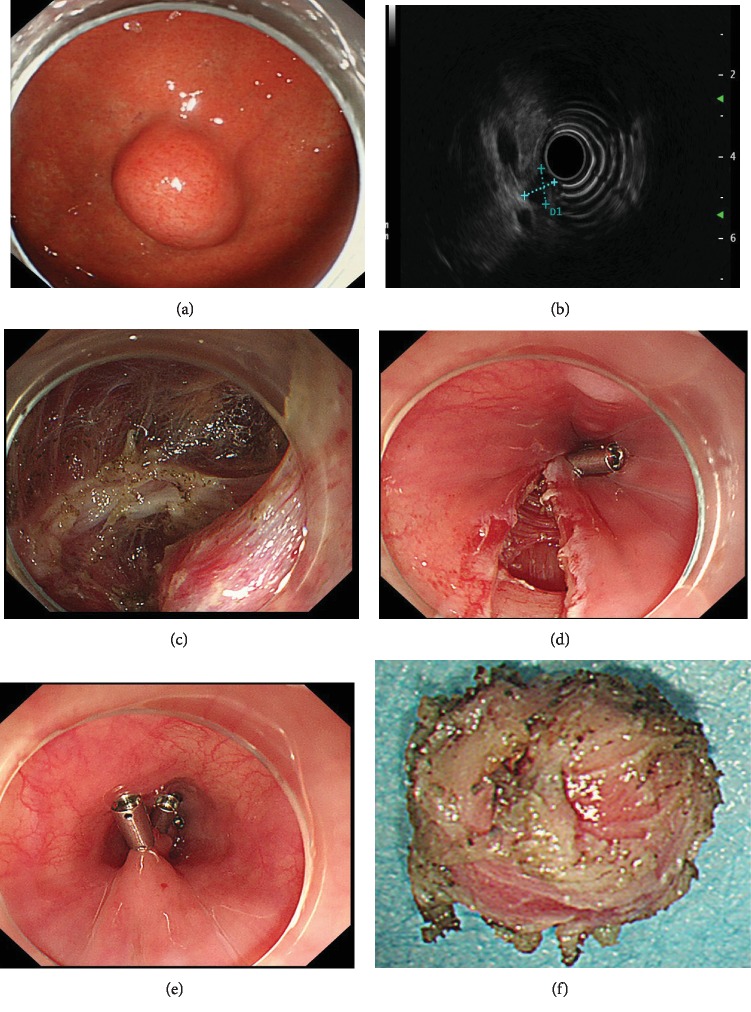
Submucosal tunneling endoscopic resection of a gastric GIST. (a) A gastric GIST is observed. (b) The tumor originates from the muscularis propria layer on EUS. (c) A submucosal tunnel was created between the submucosal and muscularis propria layer, and then the submucosal tumor was dissected. (d, e) The tunnel entry was closed using several clips. (f) View of the tumor after resection.

EUS was performed with a radial-scanning ultrasonic endoscope (GF-EU260, Olympus Co., Ltd.) to determine the layer of origin, location, and exact size of the tumor. ER was performed by two experienced specialists (T. Mao, X. Jing) using a single-channel endoscope (GIF-Q260J; Olympus Co., Ltd.). Propofol was infused for anesthesia, and the patient was kept consciously sedated with cardiorespiratory monitoring during surgery. The ESD procedures were as follows (Figure 1): First, argon plasma coagulation was used for marking at 2-3 mm from the tumor margin. An appropriate dose of indigo carmine and epinephrine was added to 0.9% normal saline and injected into the MP layer. A circumferential incision was made with an insulation-tipped (IT) knife (ITknife KD-611L, Olympus Co., Ltd.). Then, en bloc resection of the tumor from the MP layer was achieved. Bleeding was managed successfully with argon plasma coagulation and hot biopsy forceps. Metal clips (HX-610-135L, Olympus Co., Ltd.) were employed for closing the perforation. ESE was the development of ESD, and the major difference between ESD and ESE procedures was the depth of endoscopic resection. Several steps of EFTR were the same as those described in the ESD procedures. However, the lesion was completely resected, including the serosal layer. By pursing the string suture technique using a nylon band and clips, the gastric wall defect was managed, as shown in Figure 2. For STER, tunnel entry and submucosal tunnels were created, then the lesion was dissected and the tunnel entry was closed (Figure 3). LECS was performed with the cooperation of endoscopists and surgeons as previously described [11].

### 2.3. Histopathological Evaluation

The removed specimens were subjected to formalin (10%) fixation, followed by histopathological examination. Immunohistochemical staining of CD34, CD117, S-100, SMA, Ki-67, and DOG-1 was performed. By counting one thousand cells in the most active area, the labeling index (LI, %) of Ki-67 was detected. The mitotic index was calculated under 50 HPF (high-power fields), and the tumor size was recorded based on the pathological findings. The risk classification standard of GISTs refers to the consensus from the National Institutes of Health [12].

### 2.4. Follow-Up

The patients were followed up regularly. Gastroscopy was conducted 6 months after ER and annually thereafter to observe wound healing and exclude any tumor recurrence or residues. Additionally, abdominal ultrasonography and/or CT was taken yearly to exclude metastasis.

### 2.5. Statistical Analysis

All statistical analyses were performed using SPSS version 21.0 statistics software (SPSS Inc., Chicago, IL, USA). Quantitative results were expressed as the mean ± SD. *P* < 0.05 was considered statistically significant.

## 3. Results

### 3.1. Clinical Characteristics

There were 60 males and 74 females enrolled in this study who were aged 56.22 ± 8.40 years (range: 36-80 years). GISTs were symptomatic in 110 patients (82%), and abdominal discomfort and pain were most common. Of these cases, only one patient complained of hematemesis. The others were found by physical examination, and they had no specific clinical manifestations. Of the 134 GISTs, GISTs were located at the gastric fundus in 69 cases, at the corpus in 48, at the antrum in 12, and at the cardia in 5. All GISTs were originated from the MP according to the EUS findings. Metastasis was absent in all patients. The clinicopathological features of the patients are listed in Table 1.

### 3.2. Outcomes of ER

Complete resection by ER was achieved in 131 of 134 patients, among which it was achieved by ESD in 58 cases, by ESE in 43, by EFTR in 25, and by STER in 5, with the complete resection rate of 98%. In addition, GISTs of two cases were resected using LECS for the extraluminal and intraluminal growth pattern identified by preoperative endoscopic ultrasonography (EUS) and abdominal CT. One case was converted to open surgery due to the tight and wide adherence of the lesion with adjacent muscle fibers and difficulty in manipulating the endoscope. There was no tumor spillage or rupture. The mean surgical time was 59.15 ± 16.35 min (range: 39-105 min). Perforation affected 28 patients (21.4%), including intentional perforation in 25 cases (19.1%) and accidental perforation in 3 cases (2.3%). All the perforations were sealed under the endoscope, with no conversion to open surgery. Pneumoperitoneum occurred in one case after the procedure, and the case recovered after conservative treatment. In this study, there was minor bleeding in all cases, with the average blood loss of lower than 20 ml, which was well managed by endoscopic hemostasis. The average length of hospital stay was 5.50 ± 2.15 days (range: 3-10 days).

### 3.3. Pathological Characteristics and Risk Classification

The mean tumor size was 1.89 ± 1.25 cm (range: 0.5-6.0 cm). The mitotic index in one patient was over 5 mitoses/50 HPF. The results of immunohistochemistry indicated that CD117 was positive in 104 patients (78.2%), CD34 was positive in 115 (86.5%), and DOG-1 was positive in 110 (82.7%). In contrast, SMA was rare, which was positive in only 18 (13.5%) patients. S-100 was negative in all cases. The labeling index (LI, %) of Ki-67 was less than 5% in each case. Mucosal erosion of tumors was found in 2 patients. In the risk classification, 106 (79.7%) were of a very low risk, 25 (18.8%) of a low risk, and 2 (1.5%) of a moderate risk (Table 2).

### 3.4. Characteristics of Large-Size GISTs

Among these cases, there were 26 patients with large-size GISTs (>2 cm), among which 8 tumors were located at the gastric fundus, 12 at the corpus, 4 at the antrum, and 2 at the cardia. All of them achieved complete resection of the lesion. Most of the large-size GISTs were located at the gastric corpus (12/26), while most of the general GISTs were located at the fundus (69/134). The perforation rate by ESD was similar for large-size GISTs (6/26) and general GISTs (28/131) (23.1% versus 21.4%, *P* > 0.05). In addition, the surgical time, the length of hospital stay, and prognosis did not differ significantly (*P* > 0.05) (Table 3).

### 3.5. Follow-Up

Of these patients who achieved successful endoscopic resection of the tumors, 125 were followed up for ≥6 months. Two moderate-risk patients were treated with imatinib mesylate after operation. Abdominal ultrasonography and gastroscopy were performed in each patient. During the follow-up of 23 ± 8 months (range: 3-48 months), there was no recurrence, metastasis, or death.

## 4. Discussion

Most GISTs have a distinct boundary with the adjacent normal tissues, and lymph node metastasis is rare [13]. Local excision can be achieved in the majority of GISTs. The efficacy of ER has been gradually recognized for GISTs. In the past, patients with the diagnosis of GISTs were mainly treated by open surgery or laparoscopic wedge resection [14, 15]. Compared with open surgery, endoscopic therapy has great advantages in surgical time, intraoperative blood loss, and postoperative recovery [15, 16]. Laparoscopic wedge resection, as a minimally invasive procedure, has been reported to be safe and feasible for GISTs, with low morbidity, short hospital stays, and long-term disease-free survival of the patients [14]. However, it is sometimes difficult to determine the appropriate resection line, and excessive normal tissues may be removed with a laparoscope as these tumors are covered by the normal gastric wall [14]. Moreover, a postoperative stricture may be easily formed after laparoscopic surgery when lesions are located near or in the gastric cardia or pylorus [17]. In contrast, endoscopic treatment provides a clearer operative view to identify a precise resection area without needless extensive excision. Besides, ER can preserve most structures of the stomach with normal digestive physiology maintained, and patients can get a better quality of life [18, 19].

ER has been increasingly applied to gastric GISTs in recent years, and the endoscopic resection includes ESD, ESE, EFTR, and STER. Selection of the endoscopic approach is closely related to the tumor site, size, growing patterns, etc. [10]. ESD is considered as an effective treatment modality for GISTs. However, it is rather hard to dissect the tumors originating from the deep muscularis propria layer. ESE and EFTR are the development of ESD, and they can enable deep excavation [20]. STER was usually used for treating cardia GISTs, which can better protect the intactness of the mucous membrane and increase healing rate [21]. However, the technical feasibility is emphasized in most studies, but the necessity of individualized treatment is usually ignored. Compared with the published studies, our study included a larger sample size, and the patients were followed up for a longer period to assess the efficacy and safety of different endoscopic methods for the treatment of gastric GISTs. In this study, 131 gastric GISTs were removed by ER, including 58 by ESD, 43 by ESE, 25 by EFTR, and 5 by STER. The en bloc resection rate was 98% (131/134 cases). Our data demonstrated that ER is feasible, safe, and minimally invasive for the resection of gastric GISTs.

Complete excision without tumor rupture is the mainstay of treatment for GISTs [22–24]. The key of ER procedures is the success of peeling the MP layer along the edge of lesions [25]. In our study, one case failed to undergo ER, who was then converted to open surgery. The lesion was located at the gastric antrum and originated from the deeper MP layer. It was not successfully resected due to its tight and wide adherence with adjacent muscle fibers. The present study showed that the difficulty of ER procedures lay in the area connected to the MP layer of the tumor. Consistent with our findings, Bialek et al. [26] also pointed out that complete tumor removal was only related to an absent or narrow connection of tumors with the MP layer.

The most common complication is perforation when GISTs are treated by endoscopic procedures. According to the previous studies, the incidence of perforation was 0-20% [27, 28]. Perforation affected 28 patients (21.4%) in the present study, including intentional perforation and accidental perforation. In EFTR, intentional perforation is not considered a complication. When the tumor originated from the deep muscularis propria layer and adhered tightly to the serosa, EFTR may be a better choice [29, 30]. In the case of “intentional” perforation, the wound surface was closured using a nylon band together with clips by experienced endoscopists. Accidental perforations should be quickly repaired during the procedure to reduce risk of pneumoperitoneum and peritonitis. In our study, we observed that pneumoperitoneum occurred in one case after applying this technique, which recovered after conservative treatment. Therefore, EFTR is considered a safe and feasible option if performed by skilled endoscopists [31, 32].

In this study, the average length of hospital stay was 5.50 ± 2.15 days (range: 3-10 days), which was consistent with previous reports in other endoscopy centers in Asia [19, 20, 33]. In our center, before the operation, all patients were required to receive a clinical evaluation, including EUS and CT scan during hospitalization. All the patients were observed 2-4 days after ER. Abdominal signs, body temperature, and the properties of feces were strictly monitored to find and treat delayed perforation and bleeding as early as possible. In addition, the patients with intraoperative perforation were required to fast for approximately 3-4 days until abdominal pain disappeared. However, the average length of hospital stay was 5.50 days, which was longer than that after ER, laparoscopic resection, and even open resection of GISTs in many specialized centers in the US and Europe. Andalib et al. [34] reported a mean length of hospital stay of 2.08 days for the patients with GISTs after endoscopic resection in North America. The length of hospital stay seems a bit long for a minimally or less invasive procedure in our center, which may be related to cultural and ethnic differences in practice.

Usually, patients with GISTs are asymptomatic or lack the specific clinical symptoms in the early stages of these tumors. With the widespread application of endoscopic ultrasound and improved recognition of the disease, the detection rate of GISTs smaller than 2 cm in size has risen in recent years [9]. In our study, the majority of GISTs were less than 2 cm in size, while 110 patients (82%) were symptomatic with abdominal discomfort and pain most common. These symptoms were not completely alleviated in most patients following removal of GISTs. As a result, combined with our own experience and the literature reports, we suggest that most of our patients' symptoms were not truly related to the tumors [19]. It is more likely that most of the GISTs were detected incidentally during endoscopic or radiologic evaluation of patients with symptoms more likely unrelated to GISTs.

EUS and CT can be employed to assess growth patterns of the tumor and the relationship between tumor sites and MP layer, which are also used to assess the feasibility of ER [8, 9]. When the tumor is mainly convex to the enterocelia, it is difficult to perform ER [35]. It is also difficult to determine an appropriate resection line using a laparoscope, especially for intragastric and intramural GISTs [14]. A technique (LECS) that combines laparoscopic gastric resection with luminal endoscopic removal has been recommended by NCCN (National Comprehensive Cancer Network) as a treatment for gastric GIST regardless of the tumor location [36]. In our study, GISTs of two cases were resected using LECS due to the extraluminal and intraluminal growth pattern identified preoperatively by EUS and abdominal CT. This procedure was completed with simultaneous application of laparoscopic and endoscopic visualization to establish the exact borders of tumor and perform a precise resection with minimal margins [36].

In our study, 26 patients had large-size (range: 2-6 cm) GISTs, including 25 cases with tumor diameters between 2 and 5 cm and one case larger than 5 cm. All of them achieved complete resection of the lesions by ER. The perforation rate, surgical time, length of hospital stay, and prognosis did not differ significantly between large-size GISTs and general GISTs (*P* > 0.05). He et al. [37] also reported that ESD was feasible for large-size GISTs in the stomach. Hence, it seems that the tumor size is not a limiting factor for endoscopic therapy. Nevertheless, it is not easy to take out a larger tumor at the stomach via the esophagus and mouth.

The pathologic risk is an important prognostic factor for GISTs [38, 39]. In this study, most patients were of a very low risk, and only two cases were of a moderate risk based on the mitotic index and tumor size. The patients with moderate-risk GISTs were given imatinib mesylate to prevent metastasis or recurrence. During the follow-up period of 23 ± 8 months (range: 3-48 months), none of these patients had tumor recurrence and metastasis. We think it may be related with the low risk classification of patients in our study. Liang et al. [40] reported that survival of gastric GISTs patients who had Ki-67 LI ≥ 5% was shorter compared to those with Ki-67 LI < 5%. In our study, immunohistochemical analysis revealed that all 133 patients with GISTs had Ki-67 LI < 5%. We plan to analyze the long-term recurrence and survival rates of patients in the future.

There are several limitations in this study. Firstly, there are potential information biases resulting from the retrospective nature of the study. The absent randomization might lead to the selection bias. Secondly, although the sample size is relatively large, a single-center study remains a shortcoming. Finally, the follow-up was too short and the long-term results cannot be obtained from this study.

In conclusion, the results showed that ER is a feasible, effective, and safe treatment modality for gastric GISTs, including large-size GISTs. The tumor type and clinicopathological characteristics can be assessed by EUS, which guide selection of treatment modalities. LECS is recommended for intragastric and intramural GISTs. Individualized treatment of GISTs is particularly important. The efficacy and safety of ER in gastric GISTs remain to be further investigated by future prospective multicenter studies.

## Figures and Tables

**Table 1:** Characteristics of the patients and GISTs.

Age (years) (mean ± SD)	56.22 ± 8.40 (range: 36-80)
Gender, *n* (%)	
Male	60 (44.7)
Female	74 (55.3)
Symptomatic, *n* (%)	110 (82.1)
Asymptomatic, *n* (%)	24 (17.9)
Tumor site, *n* (%)	
Gastric fundus	69 (51.5)
Gastric corpus	48 (35.8)
Gastric antrum	12 (9.0)
Gastric cardia	5 (3.7)
Tumor size, *n* (%)	
≤20 mm	108 (80.6)
>20 mm	26 (19.4)
Origin (%)	
Superficial MP layer	104 (77.6)
Deeper MP layer	30 (22.4)

**Table 2:** Pathological characteristics and risk classification.

Size, *n* (%)	
<2 cm	107 (80.4)
2–5 cm	25 (18.8)
>5 cm	1 (0.75)
Mitotic index, *n* (%)	
<5/50 HPF	132 (99.2)
>5/50 HPF	1 (0.75)
Risk classification, *n* (%)	
Very low risk	106 (79.7)
Low risk	25 (18.8)
Intermediate risk	2 (1.5)

**Table 3:** Characteristics of large-size GISTs.

	Total GISTs (*n* = 134)	Large-size GISTs (*n* = 26)	*P*
Gender, *n* (%)			0.532
Male	60 (44.7)	12 (46.2)	
Female	74 (55.3)	14 (53.8)	
Tumor size	1.89 ± 1.25 cm (range 0.5-6.0)	2.9 ± 1.75 cm (range 2.0-6.0)	0.025
Tumor site, *n* (%)			0.236
Gastric fundus	69 (51.5)	8 (30.8)	
Gastric corpus	48 (35.8)	12 (46.2)	
Gastric antrum	12 (9.0)	4 (15.4)	
Gastric cardia	5 (3.7)	2 (7.7)	
Perforation during ESD, *n* (%)	28 (21.1)	6 (23.1)	0.514
Procedure time	59.15 ± 16.35 min (range: 39-105)	60.11 ± 10.21 min (range: 40-100)	0.862
Hospital stay (days)	5.50 ± 2.15 days (range 3-10)	5.80 ± 2.53 days (range 3-10)	0.791
Recurrence, *n* (%)	0 (0)	0 (0)	

## Data Availability

The retrospective data used to support the findings of this study are included within the article.
